# Perioperative Hyper-coagulation and Thrombosis: Cost Analysis After Congenital Heart Surgery

**DOI:** 10.1007/s00246-024-03554-1

**Published:** 2024-06-20

**Authors:** Puja Dutta, Meena Nathan, Sitaram M. Emani, Sirisha Emani, Juan C. Ibla

**Affiliations:** 1 Department of Cardiac Surgery, Boston Children’s Hospital, Boston, MA USA; 2 Department of Surgery, Harvard Medical School, Boston, MA USA; 3 Division of Cardiac Anesthesia, Department of Anesthesiology, Critical Care, and Pain Medicine, Boston Children’s Hospital, 300 Longwood Ave, Boston, MA 02215 USA; 4 Department of Anesthesia, Harvard Medical School, Boston, MA USA

**Keywords:** Thrombosis, Congenital heart disease, Cost, Perioperative care, Blood product conservation, Outcomes

## Abstract

Thrombosis, a major adverse event of congenital heart surgery, has been associated with poor outcomes. We hypothesized that in CHD patients undergoing cardiac surgery, increased perioperative use of pro-coagulant products may be associated with postoperative thrombosis in the setting of hyperfibrinogenemia, leading to greater hospital and blood product costs. Single-center retrospective study. Data from Boston Children’s Hospital’s electronic health record database was used in this study. All patients undergoing congenital heart surgery between 2015 and 2018 with postoperative fibrinogen levels above 400 mg/dl were reviewed. Of 334 patients with high plasma fibrinogen levels, 28 (8.4%) developed postoperative thrombosis (median age: one year, 59% male). In our cohort, 25 (7%) demonstrated evidence of baseline hypercoagulability by one or more panel test results. Thrombosis was associated with greater hospital and blood product costs, longer ventilation times, and longer hospital and ICU length of stays. Preoperative hypercoagulable state (odds ratio: 2.58, 95% CI [1.07, 9.99], p = 0.002), postoperative red blood cell transfusion (odds ratio: 1.007, 95% CI [1.000, 1.015], p = 0.04), and single ventricle physiology (univariate odds ratio: 2.94, 95% CI [1.09, 7.89], p = 0.03) were predictors of postoperative thrombosis. Preoperative hypercoagulable state and intraoperative platelet transfusion were predictors of hospital cost. Thrombosis was associated with worse in-hospital outcomes and higher costs. Preoperative hypercoagulable state and postoperative red blood cell transfusion were significant predictors of thrombosis. Risk prediction models that can guide thrombosis prevention are needed to improve outcomes of patients undergoing congenital heart surgery.

**Supplementary Information:**

The online version contains supplementary material available at 10.1007/s00246-024-03554-1.

## Introduction

Thrombosis rates in patients undergoing congenital heart surgery have increased in the current era and are continuing to rise [1, 2]. The prevalence of thrombosis in congenital heart disease patients is estimated to be 11 percent in pediatric patients undergoing congenital heart surgery and 9% in neonates and infants. [1, 3, 4] Patients undergoing congenital heart surgery are at a high risk of developing thrombosis due to disruption of blood flow, platelet activation secondary to extracorporeal circulatory support, inflammation, and/or hyper-coagulable conditions. [5, 6] Fibrinogen is a three-chain biomolecule involved in coagulation and thrombosis. [7] Hyperfibrinogenemia has been known to be associated with adverse cardiac events after cardiac surgery, [8] and the interaction of fibrinogen with red blood cells is known to promote thrombus formation and contribute to the pathophysiology of venous thromboembolism. [9]

Assuming 375,000 to 425,000 new cases per year, the estimated overall annual cost of venous thromboembolism alone is $7 to $10 billion in the United States. [10] Few studies have examined the cost of thrombosis specifically among pediatric patients with hyperfibrinogenemia undergoing congenital heart surgery. Data remains scant on whether postoperative thrombosis is a potential primary or secondary adverse event and its relationship to hospital costs in congenital heart disease (CHD) patients undergoing cardiac surgery.

The significant prevalence of postoperative thrombosis in CHD patients calls for a better understanding of the risks and complications associated with thrombosis that may in turn lead to greater hospital costs. [1, 5] Our study examines the frequency, risk factors, resource use, and outcomes associated with postoperative thrombosis in CHD patients with postoperative hyperfibrinogenemia undergoing cardiac surgery. We hypothesized that in CHD patients undergoing congenital heart surgery, increased perioperative use of pro-coagulant products may be associated with postoperative thrombosis in the setting of hyperfibrinogenemia, leading to greater hospital and blood product costs.

### Methods

## Study Data

In this retrospective descriptive study, data for 334 patients (neonates, children, adults) undergoing congenital heart surgery at Boston Children’s Hospital between 2015 and 2018 with postoperative fibrinogen levels greater than 400 mg/dl were reviewed. We excluded patients who did not have hyperfibrinogenemia, defined as fibrinogen levels greater than 400 mg/dl in the 48 h following surgery (Supplemental Fig. 1). We selected a range of 48 h post-surgery as most bleeding complications occur within the first two post-operative days. The cutoff of 400 mg/dl was selected for the fibrinogen level as this is the upper limit of the normal fibrinogen range. We selected patients with elevated plasma fibrinogen levels after surgery because these patients are at an elevated risk of developing thrombosis. [5–7, 9] Data for the study was abstracted from the institutional electronic database. Institutional Review Board approval (institutional review board approval number: P00016625, approval date: 8/15/2022) with waiver of consent was obtained for this study. The following data were collected for each patients’ index hospitalization.

**Fig. 1:**
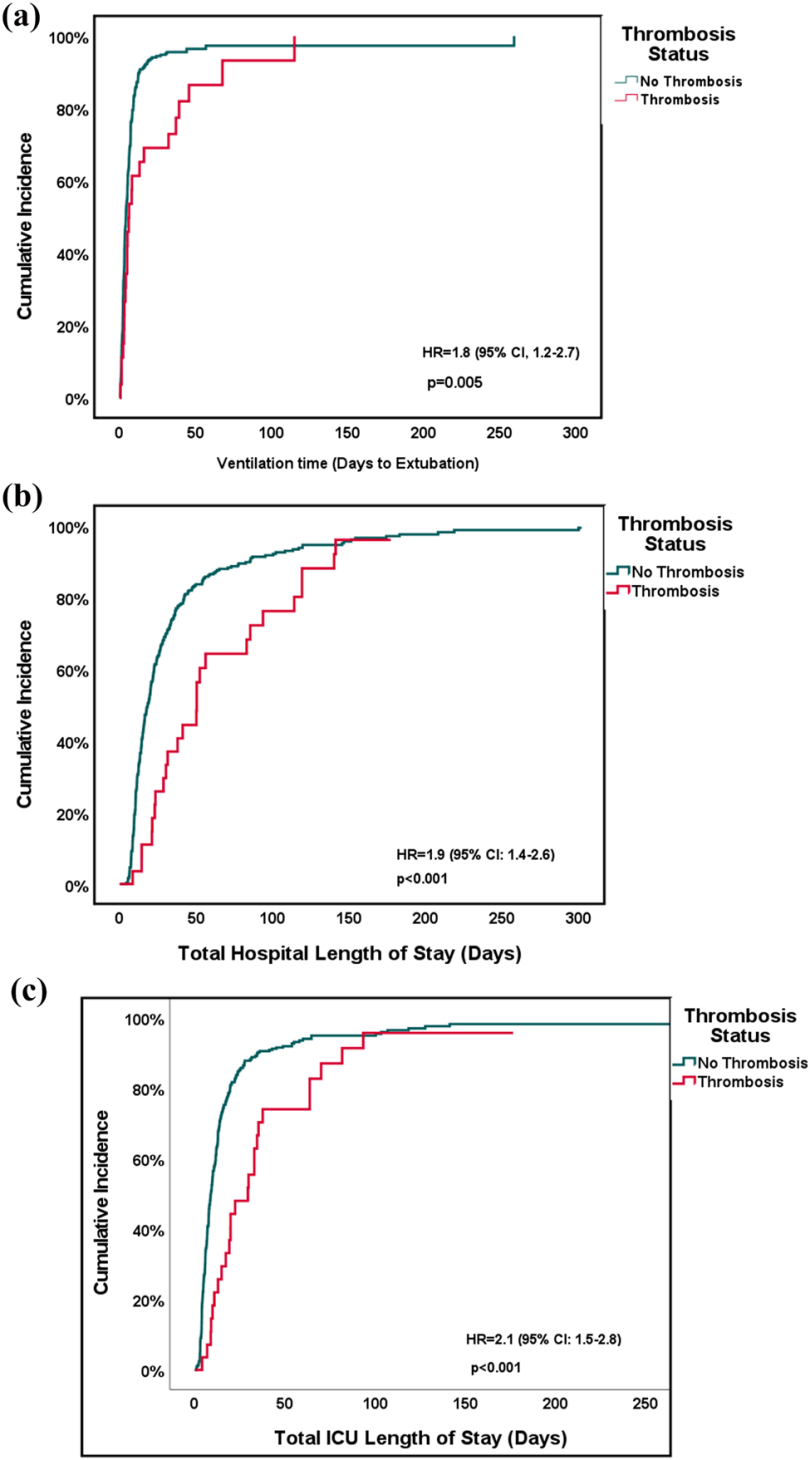
Cox proportional hazards model of thrombosis and primary outcomes. Cumulative incidence curves of Cox proportional hazards models of **a** postoperative ventilation time, **b** total hospital length of stay, and **c** total intensive care unit length of stay. Death was classified as a competing risk. The hazards ratio, 95% confidence interval, and corresponding *p*-value are also displayed

*Preoperative data:* included demographic data such as age and sex, as well as type of procedure and ventricular anatomy (single ventricle, biventricular, etc.). We included case complexity as determined by the Society of Thoracic Surgeons-European Association of Cardio-Thoracic Surgery Congenital Heart Surgery Mortality Categories (STAT), [10] preoperative anticoagulant medications, preoperative renal and hepatic dysfunction, baseline hypercoagulable panel testing, and preoperative laboratory measurements. Baseline hypercoagulability testing results were available for all patients, and the presence of any coagulopathies were noted in patient charts. We also collected prior history of stroke or thrombosis, prior history of open-heart surgery, and the number of prior sternotomies.

*Intraoperative data:* included blood product usage categorized as yes or no and quantified as ml per kg. Our approach to transfusion involves all patients receiving cell saver. A second class of patients additionally receive platelets only. A third class of patients receive cell saver, platelets, and cryoprecipitate. A fourth class of patients receive cell saver, platelets, cryoprecipitate, and any of the following: packed red blood cells, fresh frozen plasma, and/or activated factor VIIa. Our approach to transfusion has not changed significantly in the past few years [12, 13].

*Postoperative data*: included postoperative adverse events, total hospital, and intensive care unit (ICU) length of stay, ventilation time (days), blood product usage, and hospital and blood product costs. All hospital and blood product costs reported were adjusted for inflation at the time of data abstraction.

Outcomes included postoperative ventilation time, total ICU and hospital length of stay, and hospital and blood product cost. The primary predictor for these outcomes was postoperative thrombosis following the congenital heart surgery. Additionally, we explored preoperative factors associated with postoperative thrombosis.

### Definitions

*Postoperative thrombosis: Postoperative* thrombosis was defined as clinically significant postoperative thrombosis diagnosed with imaging (intravascular or intracardiac thrombus) and/or requiring therapy (anticoagulation, surgical re-intervention, catheterization). We included both partial and total venous thrombosis, arterial thrombosis, and shunt thrombosis in our definition. Early-stage thrombosis was defined if diagnosed on or before 14 days from index surgery, and thrombosis that diagnosed at any time after 14 days following index surgery was classified as late-stage thrombosis. Thrombosis data was collected for each surgical encounter until discharge from the hospital.

*STAT Categories *[10]: These categories assign the risk of mortality associated with a particular procedure based on cumulative empiric data collected in the Society of Thoracic Surgery Congenital Heart Surgery database. The risk of mortality increases as procedure complexity increases, with mortality category one having the lowest and five the highest mortality risk [14]. We further categorized these mortality categories into low risk (mortality categories 1, 2, and 3; used as reference categories in univariable and multivariable models) and high risk (mortality categories 4, 5) due to the smaller sample size studied.

*Preoperative Hypercoagulable panel tests:* These tests look for hypercoagulable states such as Factor V Leiden mutations, prothrombin gene mutations, low levels of anti-thrombin III, protein C, and protein S, and high levels of fasting plasma homocysteine, lupus anticoagulant antibodies, and anti-cardiolipin antibodies. Hypercoagulability panel testing was routinely done for all patients before surgery as part of clinical care. Given our small sample size, age-appropriate cutoffs were not used. If multiple values were present, the preoperative value closest to the time of surgery was used. We classified each test as categorical “yes or no variables” and defined overall hypercoagulable state as having one or more of these hypercoagulable conditions. As indicated by Deitcher and Chan et al., positivity in any one of these tests is considered a hypercoagulable state. We therefore created a variable “preoperative hypercoagulable state” that is positive if any one of these tests is positive [15, 16].

*Hospital costs:* were defined as the total cost amount in dollars for the hospital stay of the patient for the index hospitalization. This data was provided by the financial operations department at our institution.

*Postoperative adverse events*: included extracorporeal membrane oxygenation, ventricular assist device use, cardiac arrest, renal dysfunction requiring dialysis, re-exploration for bleeding, unplanned cardiac reoperation, unplanned cardiac catheterization intervention, unplanned non-cardiac operation, stroke, and death as defined by the Society of Thoracic Surgeons Congenital Heart Surgery Database.

### Statistical Analyses

Categorical variables are summarized as numbers and percentages (%) and continuous variables are summarized as medians and interquartile ranges. Proportions and odds ratios are presented with 95% confidence intervals.

Associations with postoperative thrombosis were examined by univariable logistic regression for categorical variables and Wilcoxon rank sum tests for continuous variables. We then used multivariable logistic regression to identify factors associated with thrombosis, using forward selection with a *p* < 0.1 inclusion criteria. Cox proportional hazards model with death as a competing risk was used for time-to-event analysis of length of stays and postoperative ventilation duration, using forward selection with a *p* < 0.1 for inclusion criteria. We also accounted for collinearity and interactions. To analyze hospital costs, we used multivariable median regression with a forward selection algorithm. A sensitivity analysis was performed to explore baseline characteristics between early and late-stage thrombosis groups as well as any differences in blood products transfused intraoperatively. Chi-square and Wilcoxon rank sum tests were used for categorical and continuous variables, respectively.

A *p-*value of 0.05 or less was considered to be statistically significant. We analyzed all data using SAS version 9.4 (SAS Institute Inc., Cary, NC).

## Results

### Patient Characteristics

Among 3,259 patients who had congenital heart surgery during the study time frame, 334 patients (10%) had postoperative hyperfibrinogenemia within 48 h after surgery. Of 334 patients in our cohort, 28 (8.4%) developed thrombosis postoperatively. The types of thrombosis (e.g., arterial, venous, etc.) and their sites for all patients with postoperative thrombosis are summarized in Supplemental Table 1. Overall, there were 195 (59%) males, and the median age was one (IQR: 0–8) years. In our cohort, 25 (7.5%) patients were hypercoagulable on one or more hypercoagulable panel tests, with antithrombin III deficiency being the most common hypercoagulable condition: 12 (4%) patients in the no thrombosis group versus 5 (18%) patients in the thrombosis group (p = 0.0009). Table 1 provides a summary of baseline characteristics for all patients in our cohort.

**Table 1:** Baseline characteristics of all patients undergoing congenital heart surgery (CHD) (N = 334)

Characteristic	All Cases	No thrombosis	Postoperative thrombosis	*P* value
N (%)	334 (100)	306 (91.6)	28 (8.4)	
Age (years)	1 (0–8)	1 (0–7)	1.5 (0–13.5)	0.61
Weight (kg)	8.9 (4.0–20.5)	8.6 (4.0–19.2)	11.5 (3.9–44.6)	0.48
Gender				
Female	138 (41)	131 (43)	7 (25)	0.06
Male	195 (59)	174 (57)	21 (75)	
**Cardiac Anatomy**				0.26
Single ventricle	32 (10)	26 (9)	6 (21)	
Biventricular	288 (86)	267 (87)	21 (75)	
BiV conversion	10 (3)	9 (3)	1 (3)	
BiV staging	2 (0.6)	2 (0.6)	0 (0)	
BiV recruit	2 (0.6)	2 (0.6)	0 (0)	
STAT mortality category				0.005^a^
0	11 (3)	7 (2)	4 (14)	
1	13 (4)	13 (4)	0 (0)	
2	73 (22)	69 (23)	4 (11)	
3	60 (18)	58 (19)	2 (7)	
4	131 (39)	116 (38)	15 (54)	
5	45 (14)	42 (14)	3 (11)	
Surgical procedure				
AVV repair/replacement	97 (29)	92 (30)	5 (18)	
Pulmonary valve/RV-PA conduit	63 (19)	59 (19)	4 (14)	
Neo/Aortic repair/replacement	42 (13)	39 (13)	3 (11)	
Stage 1	27 (8)	25 (8)	2 (7)	
TOF repair	26 (8)	22 (7)	4 (14)	
Septal Defects	22 (6)	22 (7)	2 (7)	
Transplant	15 (4)	15 (5)	0 (0)	
Neo/Aortic root surgery	8 (2)	8 (3)	0 (0)	
Fontan revisions	9 (3)	7 (2)	2 (7)	
Pacemaker/arrhythmia surgeries	10 (3)	7 (2)	3 (11)	
Cone	7 (2)	6 (2)	1 (4)	
Other	6 (2)	4 (1)	2 (7)	
AAOCA/coronary surgeries	2 (0.6)	2 (0.7)	0 (0)	–
Preoperative anticoagulants	129 (38)	114 (37)	15 (54)	0.09
Aspirin	95 (28)	85 (28)	10 (36)	0.37
Clopidogrel	4 (1)	3 (1)	1 (3)	0.30
Coumadin	17 (5)	16 (5)	1 (3)	1.00
Enoxaparin sodium	11 (3)	11 (4)	0 (0)	0.61
IV Heparin	28 (8)	23 (8)	5 (18)	0.07^b^
NoAC	2 (0.6)	1 (0.3)	1 (3)	0.16
Dabigatran	1 (0.3)	0 (0)	1 (3)	0.08^b^
Rivaroxaban	1 (0.3)	1 (0.3)	0 (0)	1.00
Preoperative comorbidities				
Liver dysfunction	11 (3)	10 (3)	1 (3)	1.00
Renal dysfunction	18 (5)	15 (5)	3 (11)	0.18
Hypercoagulable states	25 (7)	18 (6)	7 (25)	< 0.001^a^
Anti-thrombin III deficiency	17 (5)	12 (4)	5 (18)	0.009^a^
Protein C Activity	1 (0.3)	1 (0.3)	0 (0)	1.000
Protein S Activity	1 (0.3)	0 (0)	1 (3)	0.08
Lupus anticoagulant	2 (0.6)	2 (0.7)	0 (0)	1.00
Anti-cardiolipin	4 (1.2)	4 (1.3)	0 (0)	1.00
Pts with one hypercoagulable state	24 (7)	16 (5)	8 (29)	< 0.001^a^
Pts with two hypercoagulable states	1 (0.3)	0 (0)	1(3)	0.08
Pts with > 2 hypercoagulable state	0 (0)	0 (0)	0 (0)	1.00
Prior Sternotomies	93 (28)	22 (9)	6 (6)	0.42
Number Prior Sternotomies	0 (0–2)	0 (0–2)	0 (0–1)	0.63
Preoperative coagulation tests				
PT	14.4 (13.4–16)	14.4 (13.4–16.0)	14.2 (13.4–15.8)	0.74
PTT	35.7 (30.9–43.9)	35.1 (30.7–42.2)	38.2 (33.6–71.4)	0.04^a^
INR	1.1 (1.0–1.3)	1.1 (1.0–1.3)	1.1 (1.0–1.3)	0.68
Hematocrit	39.9 (35.8–44.9)	40.0 (35.8–44.9)	37.9 (34.6–44.0)	0.20
Hematocrit (post op)	37.4 (33.6- 41.3)	37.3 (33.7–41.4)	38.6 (32.9–41.3)	0.85
Preoperative creatinine	0.4 (0.26–0.6)	0.4 (0.3–0.6)	0.37 (0.26–0.7)	0.79
Postoperative fibrinogen^*^ (mg/dL)	493 (441–556.5)	496 (441–559.5)	472 (425–520)	0.20
Products given in operating room				
Platelets				
N (%)	244 (73)	221 (72)	23 (82)	0.25
Volume (mL/kg)	13.33 (0–25.9)	13.2 (0–25.7)	17.4 (5.6–35.4)	0.16
Combined Platelets^†^				
N (%)	280 (83)	256 (84)	24 (86)	1.00
Volume (mL/kg)	18.3 (9.3–30.2)	18.2 (9.6–30.0)	19.3 (7.6–35.4)	0.61
Cryoprecipitate				
N (%)	173 (52)	156 (51)	17 (61)	0.32
Volume (mL/kg)	2.0 (0–13.6)	1.6 (0–13.6)	3.9 (0–13.1)	0.63
Products given in operating room (cont.)				
Plasma				
N (%)	15 (5)	15 (5)	0 (0)	0.23
Volume (mL/kg)	21 (14–34)	21 (14–34)	-	–
RBCs				
N (%)	68 (20)	59 (19)	9 (32)	0.11
Volume (mL/kg)	0 (0–0)	0 (0–0)	0 (0–6.7)	0.16
Cell Saver				
N (%)	295 (88)	271 (88)	24 (86)	0.55
Volume (mL/kg)	18.4 (9.4–32.9)	19.1 (9.7–32.6)	14.3 (6.6–37.1)	0.44
Concentrated platelets^‡^				
N (%)	56 (17)	51 (17)	5 (18)	0.80
Volume (mL/kg)	0 (0–0)	(0–0)	0 (0–0)	0.95
Factor VIIa				
N (%)	52 (16)	46 (15)	6 (21)	0.37
Volume (mL/kg)	0 (0–0)	0 (0–0)	0 (0–0)	0.34
Products given in ICU				
Platelets				
N (%)	51 (15)	46 (15)	5 (19)	0.63
Volume (mL/kg)	0 (0–0)	0 (0–0)	0 (0–0)	0.55
Cryoprecipitate				
N (%)	41 (12)	35 (11)	6 (22)	0.10
Volume (mL/kg)	0 (0–0)	0 (0–0)	0 (0–0)	0.10
RBCs				
N (%)	176 (53)	159 (52)	17 (61)	0.37
Volume (mL/kg)	5.0 (0–15.4)	4.2 (0–15.2)	10.4 (0–20.7)	0.20
Plasma				
N (%)	55 (17)	47 (15)	8 (29)	0.07
Volume (mL/kg)	0 (0–0)	0 (0–0)	0 (0–8)	0.06
Major Complications	95 (29)	76 (25)	19 (68)	
Number of Complications	0 (0–1)	0 (0–0)	1(0–2.5)	
Renal Failure requiring dialysis	4 (1)	2 (0.6)	2 (7)	
Cardiac Failure	36 (11)	30 (10)	6 (21)	
ECMO/VAD	22 (7)	17 (6)	5 (18)	
Re-exploration for bleeding	10 (3)	8 (3)	2 (7)	
Unplanned cardiac catheterization	18 (5)	14 (5)	4 (14)	
Death	10 (3)	7 (2)	3 (11)	
MSOF	1 (0.3)	0 (0)	1 (4)	
Stroke	7 (2)	3 (1)	4 (14)	
Unplanned non-cardiac reoperation	3 (0.9)	2 (0.67)	1 (4)	
Unplanned event reoperation	8 (2)	0 (0)	8 (29)	

*Postoperative fibrinogen levels are 48 h post-surgery

^a^Statistically significant

^†^Combined platelets refer to platelets and concentrated platelets composite value combined for analysis

^‡^Concentrated platelets are a plasma reduced preparation of platelets

*BiV* biventricular, *STAT* Society of Thoracic Surgeons- European Association of Cardio-Thoracic Surgery Congenital Heart Surgery Mortality Categories; *AVV* atrioventricular valve; *RV-PA* right ventricle-pulmonary artery; *AAOCA* anomalous aortic origin of a coronary artery; NOAC: novel oral anticoagulants; *PT* prothrombin; *PTT* partial thromboplastin time; *INR* internal normalized ratio;; *ml* milliliter; *kg* kilogram; *RBCs* red blood cells, *ICU* intensive care unit; *ECMO* extracorporeal membrane oxygenation, *VAD* ventricular assist device, *MSOF* multi-system organ failure

### Postoperative Adverse Events

The most common adverse events in our cohort were cardiac failure (defined as cardiac arrest or dysfunction) (11%), the need for postoperative mechanical circulatory support (7%), and unplanned catheter re-intervention (5%). Overall, seven patients (2%) experienced stroke and 10 patients (3%) died within the hospital or within 30 days after discharge.

### Time to Diagnosis

The median time to thrombosis diagnosis was 13 (interquartile range: 3.5–30) days. A sensitivity analysis revealed no significant differences between early and late-stage thrombosis patients in baseline characteristics and products transfused in the operating room.

### Outcomes: Hospital and Intensive Care Unit Length of Stay, Ventilation Time (Days), and Cost

Table 2 summarizes the outcomes of cost, ventilation time, and length of stay in our cohort. Patients who developed thrombosis postoperatively experienced significantly greater hospital costs (median: $293,712 interquartile range: [$193,971, $702,250]) compared to those without thrombosis (median: $135,045 interquartile range: [$89,048, $245,865] p < 0.001). Figure 1 displays the cumulative incidence curves for hospital length of stay, intensive care unit length of stay, and ventilation time. Supplemental Tables 2–4 show the results of univariable cox regression with postoperative thrombosis as the primary predictor and death as a competing risk for these three outcomes.

**Table 2:** In-hospital outcomes for all patients (N = 334)

	No Thrombosis (n = 306)	Postoperative Thrombosis (n = 28)	*P* value
Cost (dollars)			
Hospital Cost	135,045 (89,048, 245,865)	293,712 (193,971, 702,250)	< 0.001^a^
Blood Product Cost	3298 (2124, 5469)	8917 (4,130, 13,280)	< 0.001^a^
Length of Stay (days)			
Total Hospital LOS	18.17 (10.40, 35.29)	49.20 (23.05, 89.43)	< 0.001^a^
Postoperative Hospital LOS	16.21 (9.37, 30.04)	43.05 (22.13, 87.49)	< 0.001^a^
Total CICU LOS	8.88 (4.83, 16.06)	25.92 (11.87, 42.72)	< 0.001^a^
Postoperative CICU LOS	7.57 (4.69, 13.55)	22.2 (11.87, 41.56)	< 0.001^a^
Ventilation Time (days)	4.16 (2.23–7.28)	6.01 (3.01, 35.22)	0.03^a^

^a^Statistically significant

*CICU* cardiac intensive care unit; *LOS* length of stay

Multivariable cox regression (with death as a competing risk) of postoperative thrombosis and perioperative factors associated with longer length of stay and ventilation time are presented in Supplemental Tables 5 and 6. Postoperative thrombosis was an independent predictor of all three time variables: hospital length of stay (hazard ratio: 2.26 days, 95% confidence interval: [1.48, 3.44] days, p < 0.001), intensive care unit length of stay (hazard ratio: 2.58 days, 95% confidence interval: [1.69, 3.93] days, p < 0.001), and ventilation time (hazard ratio: 1.81 days, 95% confidence interval: [1.18, 2.79] days, p = 0.007).

### Risk Factors Associated with Postoperative Thrombosis

On univariable analysis, single ventricle physiology was significantly associated with postoperative thrombosis (odds ratio: 2.94, 95% confidence interval: [1.09, 7.89], p = 0.03). In a multivariable model, we found overall hypercoagulable state (odds ratio: 2.58, 95% confidence interval: [1.07, 9.99], p = 0.002) and postoperative red blood cell transfusion (odds ratio: 1.007, 95% confidence interval: [1.000, 1.015], p = 0.04) to be independent predictors of postoperative thrombosis (Table 3). The c-statistic for this model was 0.69. There was no significant association between increased fibrinogen levels in our cohort and risk of thrombosis.

**Table 3:** Univariable and multivariable logistic regression of parameters with thrombosis (N = 334, Max re-scaled R^2^ = 0.09, c-statistic = 0.69)

Parameters	Univariable		Multivariable	
	OR (95% CI)	*P* value	OR (95% CI)	*P* Value
Age (months)	1.00 (0.99, 1.00)	0.77	–	–
STAT mortality category 4 and 5	1.68(0.75, 3.75)	0.21	–	–
Chromosomal abnormality	1.52 (0.55, 4.24)	0.42	–	–
Cardiopulmonary bypass (min)	1.00 (0.99, 1.00)	0.78	–	–
Single Ventricle (y/n)	2.94 (1.09, 7.89)	0.03^a^	2.58 (0.90, 7.38)	0.07
Prior stroke	1.23 (0.27, 5.60)	0.79	–	–
Prior thrombosis	0.87 (0.19, 3.86)	0.85	–	–
Overall Hypercoagulable state (y/n)	5.33 (2.00, 14.20)	< 0.001^a^	3.27 (1.07, 9.99)	0.01^a^
Overall preoperative anticoagulant	1.94 (0.89, 4.23)	0.09	–	–
Overall preoperative NOAC	11.30 (0.69, 185.69)	0.08	–	–
Preoperative aspirin	1.44 (0.64, 3.26)	0.37	–	–
Preoperative clopidogrel	3.74 (0.38, 37.21)	0.26	–	–
Preoperative coumadin	0.67 (0.09, 5.26)	0.70	–	–
Preoperative IV Heparin	2.68 (0.93, 7.70)	0.07	–	–
Preoperative hepatic dysfunction	1.10 (0.14, 8.89)	0.93	–	–
Preoperative renal dysfunction	2.33 (0.63, 8.59)	0.20	–	–
Platelets in operating room (Y/N)	1.17 (0.39, 3.52)	0.78	–	–
Platelets in operating room (ml/kg)	1.00 (0.99, 1.02)	0.72	–	–
Cryoprecipitate in operating room (Y/N)	1.49 (0.67, 3.28)	0.32	–	–
Cryoprecipitate in operating room (ml/kg)	1.00 (1.00,1.00)	0.88	–	–
Cell Saver in operating room (Y/N)	0.78 (0.25, 2.36)	0.65	–	–
Cell Saver in operating room (ml/kg)	1.00 (1.00, 1.01)	0.85	–	–
RBCs in operating room (Y/N)	1.98 (0.85, 4.61)	0.11	–	–
RBCs in operating room (ml/kg)	1.00 (1.00, 1.02)	0.65	–	–
Factor VIIa in operating room (Y/N)	1.54 (0.59, 4.01)	0.37	–	–
Prior sternotomies	0.68 (0.27, 1.74)	0.43	–	–
Number prior sternotomies	0.96 (0.69, 1.35)	0.83	–	–
Platelets in ICU (Y/N)	1.29 (0.46, 3.56)	0.63	–	–
Platelets in ICU (ml/kg)	1.02 (1.00, 1.05)	0.05	–	–
Cryoprecipitate in ICU (Y/N)	2.21 (0.84, 5.86)	0.11	–	–
Cryoprecipitate in ICU (ml/kg)	1.03 (1.00, 1.06)	0.06	–	–
Plasma in ICU (Y/N)	2.20 (0.92, 5.30)	0.08	–	–
Plasma in ICU (ml/kg)	1.01 (1.00, 1.03)	0.05	–	–
RBC in ICU (Y/N)	1.43 (0.65, 3.15)	0.37	–	–
RBC in ICU (ml/kg)	1.00 (1.00, 1.01)	0.03^a^	1.00 (1.000, 1.01)	0.04^a^

^a^Statistically significant

*CI* confidence interval; *ICU* intensive care unit; *N* no; *NOAC* novel oral anticoagulant; *OR* odds ratio; *STAT* Society of Thoracic Surgeons- European Association of Cardio-Thoracic Surgery Congenital Heart Surgery Mortality Categories; *RBCs* red blood cells, *Y* yes

### Thrombosis and Hospital Cost

**Fig. 2:**
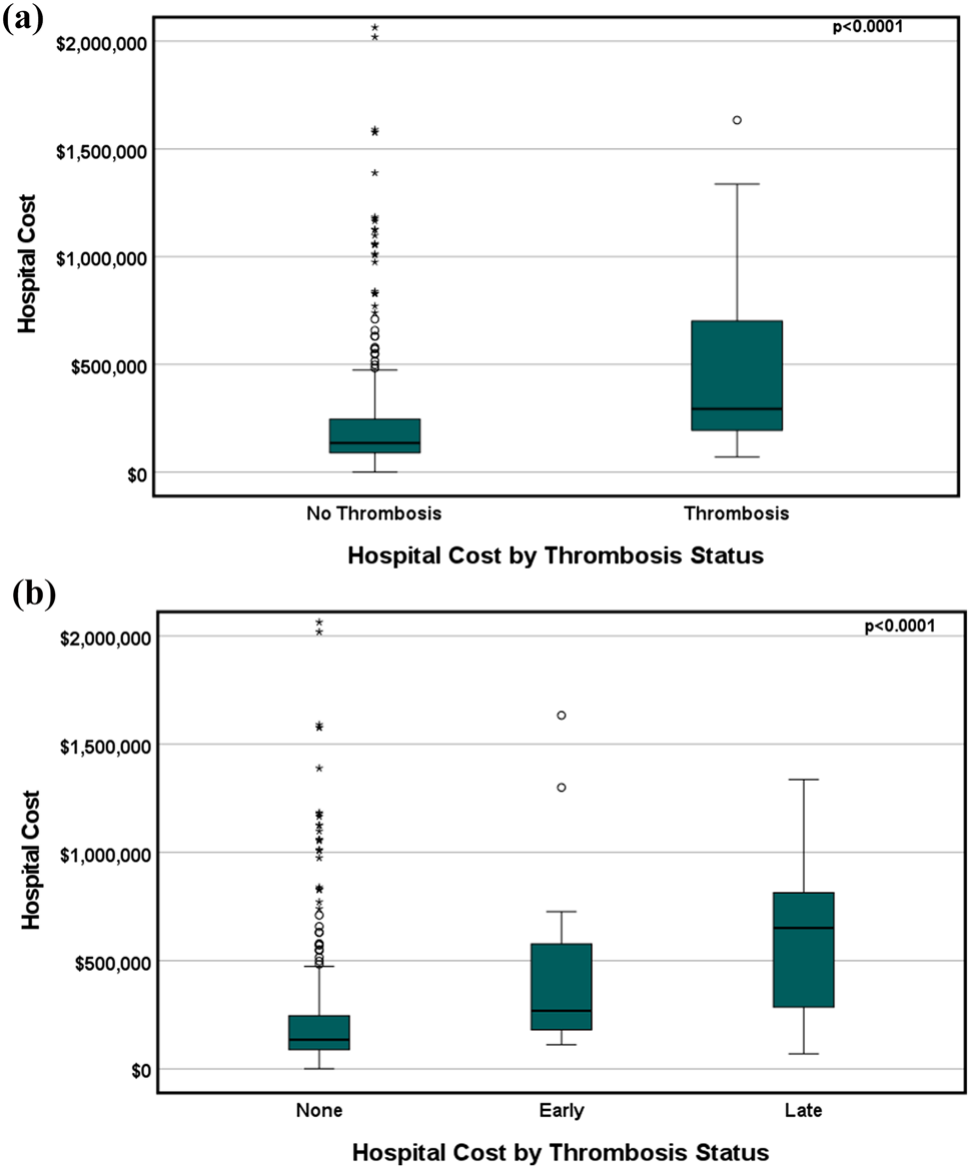
Box and whisker plots of hospital cost by thrombosis. Hospital cost by **a** Thrombosis status (yes/no) and **b** Thrombosis time category (none, early, late). The *p*-value of ANOVA is also displayed. The lower and upper borders of the box represent the 25th and 75th percentiles, the middle horizontal line represents the median, and the lower and upper whiskers represent the minimum and maximum values of non-outliers. Extra dots represent outliers

In a multivariable model (Table 4), thrombosis was related to higher hospital cost (estimate: $151,323, 95% confidence interval: [− $146, $302,792], p = 0.05). Overall hypercoagulable state (estimate: $172,829, 95% confidence interval: [$86,654, $259,004], p < 0.001) and greater intraoperative transfusion of platelets (ml/kg) (estimate: $1,640, 95% confidence interval: [$584, $2,697], p = 0.002) were both independent predictors of greater hospital cost. Prior history of thrombosis was related to hospital costs (estimate: $182,608, 95% confidence interval: [− $12,708, $377,924], p = 0.07). The adjusted R^2^ for this model was 0.17. Figure 2 displays the hospital cost by thrombosis status as a binary yes/no variable as well as classified by time to diagnosis. In accordance with Fig. 2, univariable median regression revealed later stage thrombosis as a significant predictor of hospital cost (estimate: $516,522, 95% confidence interval: [$175,809, $857,235], p = 0.003) (Supplemental Table 7).

**Table 4:** Multivariable median regression for factors associated with hospital cost (adjusted R^2^ = 0.17)

Parameter	Estimate	95% CI	*P* value
Thrombosis (y/n)	151,323	(− 146, 302,792)	0.05
Prior thrombosis	182,608	(− 12,708, 377,924)	0.07
Platelets in operating room (ml/kg)	1,640	(584, 2,697)	0.01^a^
Overall hypercoagulable state	172,829	(86,654, 259,004)	< 0.001^a^

^a^Statistically significant

*CI* confidence interval; *n* no; *y* yes

## Discussion

The frequency, risk factors, and resource use of postoperative thrombosis in the setting of hyperfibrinogenemia in the congenital heart population and its relation to hospital costs have not been well characterized. While postoperative thrombosis in CHD patients undergoing congenital heart surgery remains a significant adverse event, few studies have examined the cost of postoperative thrombosis in CHD patients with postoperative hyperfibrinogenemia. We sought to determine the risk factors for postoperative thrombosis in the congenital heart population and its subsequent effect on hospital and blood product costs.

Several studies have reported thrombosis to be one of the most common complications of cardiac surgery associated with longer hospital length of stay and significant morbidity [6, 7]. The incidence and economic burden of thrombosis is expected to increase over time [2, 17, 18]. Silvey et al. note that thrombosis rates in CHD patients after cardiac surgery is increasing, while Fernandez et al. state that the cost of thrombosis is increasing faster than general inflation for medical care services [2, 17]. These trends call for a better understanding of the risk factors that incite thrombosis, timing of postoperative thrombosis, and its effect on outcomes in the congenital heart population. Known risk factors for thrombosis in the congenital heart population include age less than 28 days, single ventricle physiology, an inflammatory state evidenced by elevated preoperative C-reactive protein levels, and elevated preoperative von Willebrand factor activity [1, 13–17, 19–22].

We found the frequency of thrombosis among patients undergoing congenital heart surgery with elevated postoperative fibrinogen levels at our institution was 8.4%. Other studies have found thrombosis rates of 6.2% in pediatric patients with congenital heart disease undergoing cardiac surgery [23]. The rate of thrombosis is as high as 20% in neonates undergoing complex cardiac surgery with hypercoagulable conditions [24].

In our study, we demonstrated that thrombosis in the setting of postoperative hyperfibrinogenemia was associated with longer ventilation times and lengths of stay, resulting in significant hospital and blood product costs. Adjusted analysis showed that overall hypercoagulable state and transfusion of red blood cells postoperatively were independent predictors of postoperative thrombosis, demonstrating that hypercoagulability measured both preoperatively and postoperatively leads to major complications of thrombosis. In a multivariable model, overall hypercoagulable state and intraoperative platelet transfusion were independent predictors of greater hospital cost. These results emphasize overall hypercoagulable state as an important risk factor for thrombosis and a need for alternative blood product utilization strategies that minimize the risk of postoperative thrombosis.

### Hypercoagulable States and Thrombosis

The hypercoagulable states seen in our study population (antithrombin III deficiency, protein C activity, protein S activity, lupus anticoagulant, and anticardiolipin antibodies) represent disruptions in the normally highly regulated coagulation cascade. These disorders have been linked to an elevated risk of arterial and venous thrombosis [15, 16, 25].

### Hyperfibrinogenemia and thrombosis

Several studies have examined hyperfibrinogenemia in the perioperative setting to assess the risk of adverse outcomes. A study by Shu et al. examines the association between hyperfibrinogenemia and severity of tumor stage, tumor metastasis, and overall survival in patients with gallbladder cancer who underwent surgical resection [26]. Another study by Wu et al. examines hyperfibrinogenemia as a marker for predicting perforated appendicitis in children [27]. Harr’s group found that postinjury hyperfibrinogenemia in patients at an academic level I trauma center surgical intensive care unit compromised heparin efficacy for VTE prophylaxis [28]. Hyperfibrinogenemia has been implicated in thrombotic events [5–7, 9], but few have investigated its role in the cost of postoperative thrombosis in patients undergoing congenital heart surgery.

Our finding that patients with hyperfibrinogenemia may have increased risk of thrombosis in the setting of antithrombin III deficiency may warrant further study. Antithrombin III inhibits thrombin as well as free factors of coagulation, including factor VIIa, IXa, and Xa [29]. Deficiency of antithrombin III is known to be a risk factor for thrombosis [29] as well as heparin resistance [30]. However, given that our study was retrospective in nature, causality between antithrombin III deficiency and thrombosis cannot be established.

Fibrinogen is a positive acute phase reactant, meaning it is upregulated and its concentration increases during inflammation [31]. Therefore, postoperative hyperfibrinogenemia seen in our cohort may be a result of a potential inflammation reaction in response to cardiac surgery. The renin–angiotensin–aldosterone system (RAAS) is known to increase IL-6 production during inflammation, and high fibrinogen levels increase IL-6 synthesis, thus exacerbating inflammation. Various nonspecific activators including surgical trauma, reperfusion injury, cardiopulmonary bypass, and blood transfusion can trigger a systemic inflammatory response in patients following surgery [32]. The inflammatory response following cardiac surgery includes the activation platelets, pro-inflammatory cytokines and chemokines, the complement system, and the coagulation pathway [32–34]. As part of the activation of the inflammatory response, fibrinolysis is inhibited, causing inadequate fibrin removal and contributing to microvascular thrombosis [35]. Further study on the relationship between fibrinogen, inflammation, and thrombosis is needed to identify therapies that target both coagulation and inflammation to prevent thrombotic complications [35].

## Limitations

The limitations of this study include those inherent to a single-center retrospective study, such as small sample size, which resulted in wide estimates of effect and restricted the number of predictors in multivariable models. A larger sample size may strengthen the power of some of our models. There are likely, also other variables involved in thrombosis risk not investigated in our study. One such risk factor, among others, is central venous catheter use [36]. Additionally, associations between thrombosis and major complications after surgery could not be analyzed for all thrombosis patients, as a proportion of them occurred either before thrombosis and were therefore in the causal pathway, or after thrombosis. The time relationship between adverse events and thrombosis was not consistent from patient to patient, making it difficult to draw any causal relationships regarding all patients with thrombosis and major complications. Furthermore, we limited our cohort to patients with elevated postoperative fibrinogen levels, as hyperfibrinogenemia is independently associated with an increased risk of thrombosis [7–9, 11, 37]. It is likely that our 400 mg/dl cutoff for postoperative fibrinogen levels preselected patients in whom hypercoagulable tests were routinely done. Given the retrospective nature of the study, the search for all patients who had thrombosis would be quite involved, and data on thrombosis was not always available in the medical record to make significant conclusions. Additionally, only patients in whom thrombosis was detected based on clinical suspicion or imaging could be included. Thus, the true incidence of thrombosis in our study may be underrepresented. Future larger studies that compare thrombosis rates using age-matched and case complexity-matched controls are needed to better quantitate the incidence and scope of this important complication.

## Conclusion

Thrombosis is a known adverse event of congenital heart surgery associated with worse outcomes. The frequency of thrombosis among patients undergoing congenital heart surgery with elevated postoperative fibrinogen levels at our institution was 8.4%. Preoperative hypercoagulable state and postoperative RBC transfusion were identified as risk factors for thrombosis. Hypercoagulable state and exposure to platelets were predictors of greater hospital cost. We demonstrated that thrombosis in the setting of perioperative hypercoagulation was associated with worse in-hospital outcomes including ventilation time and length of stay, and late-stage thrombosis was associated with greater hospital and blood product costs. In the future, standardizing protocols for imaging in high-risk patients may aid early detection and guide development of preventative strategies in the congenital heart population.

## Supplementary Information

Below is the link to the electronic supplementary material.

Supplementary file1 (DOCX 84 KB)
