# Supplementary material for: Perioperative Hyper-coagulation and Thrombosis: Cost Analysis After Congenital Heart Surgery
Source: Pediatr Cardiol. 2024 Jun 20;46(5):1328–39. doi: 10.1007/s00246-024-03554-1 (PMC12021692; doi:10.1007/s00246-024-03554-1)
Supplement: Supplementary file 1 — Supplementary file1 (DOCX 84 KB) [file 246_2024_3554_MOESM1_ESM.docx]

**Supplemental Table 1. Types of thrombosis for all patients with postoperative thrombosis (N=28)**

| **Thrombosis Site** | **Number of Patients^*^ (N=28)** |
| --- | --- |
| Arterial  Proximal femoral artery  Common femoral artery  External iliac artery  Systemic-to-pulmonary artery shunt  Left-anterior descending artery | 1 (3.5)  1 (3.5)  1 (3.5)  1 (3.5)  1 (3.5) |
| Venous  Basilic vein  Pulmonary vein  Upper extremity (not specified)  Internal jugular vein  Central vein  External iliac vein  Innominate vein  Lower extremity (not specified)  PICC line-related venous | 2 (7)  1 (3.5)  2 (7)  5 (18)  1 (3.5)  3 (11)  1 (3.5)  2 (7)  1 (3.5) |
| Cardiac  Left ventricular apex  LVAD  Sub-aortic  Aortic  Fontan  RV-PA conduit  Melody transcatheter pulmonary valve | 2 (7)  1 (3.5)  1 (3.5)  1 (3.5)  1 (3.5)  1 (3.5) 1 (3.5) |
| *^*^Some patients had multiple types of thrombosis.*  *LVAD: left-ventricular assist device; PICC: peripherally inserted central catheter: RV-PA: right ventricle-pulmonary artery;* | |

**Supplemental Table 2. Univariate cox regression of factors and hospital length of stay**

| **Parameter** | **Hazards Ratio** | **95% Confidence Interval** | ***P* value** |
| --- | --- | --- | --- |
| Thrombosis (y/n) | 1.9 | (1.4, 2.6) | <0.001 ^a^ |
| Age (months) | 1.0 | (1.0, 1.0) | <0.001 ^a^ |
| STAT mortality category 4 and 5 | 1.1 | (0.8, 1.3) | 0.62 |
| Chromosomal abnormality | 1.1 | (0.8, 1.5) | 0.38 |
| Cardiopulmonary bypass (min) | 1.0 | (0.9, 1.0) | 0.35 |
| Single Ventricle (y/n) | 1.5 | (1.0, 2.1) | 0.03 ^a^ |
| Prior stroke | 1.0 | (0.7, 1.6) | 0.86 |
| Prior thrombosis | 2.0 | (1.3, 3.1) | 0.001 ^a^ |
| Overall Hypercoagulable state (y/n) | 1.8 | (1.3, 2.5) | <0.001 ^a^ |
| Overall preoperative anticoagulant | 1.3 | (1.0, 1.6) | 0.03 ^a^ |
| Overall preoperative NOAC | 0.6 | (0.2, 1.5) | 0.32 |
| Preoperative aspirin | 1.0 | (0.8, 1.4) | 0.62 |
| Preoperative clopidogrel | 0.9 | (0.4, 2.0) | 0.78 |
| Preoperative enoxaparin sodium | 0.9 | (0.6, 1.5) | 0.82 |
| Preoperative coumadin | 0.7 | (0.4, 1.0) | 0.06 |
| Preoperative IV Heparin | 3.4 | (2.3, 4.7) | <0.001 ^a^ |
| Preoperative dabigatran | 1.0 | (0.9, 1.2) | 0.59 |
| Preoperative rivaroxaban | 0.2 | (0.2, 0.3) | <0.001 ^a^ |
| Preoperative hepatic dysfunction | 1.1 | (0.5, 2.5) | 0.80 |
| Preoperative renal dysfunction | 2.0 | (1.2, 3.2) | 0.01 ^a^ |
| Combined Platelets in OR (y/n)^*^ | 2.0 | (1.4, 2.8) | <0.001 ^a^ |
| Combined Platelets in OR (ml/kg)^*^ | 0.9 | (0.9, 1.0) | <0.001 ^a^ |
| Cryoprecipitate in OR (y/n) | 1.3 | (1.0, 1.6) | 0.02 ^a^ |
| Cryoprecipitate in OR (ml/kg) | 1.0 | (1.0, 1.0) | <0.001 ^a^ |
| Cell Saver in OR (y/n) | 0.8 | (0.5, 1.1) | 0.17 |
| Cell Saver in OR (ml/kg) | 0.9 | (0.9, 1.0) | 0.001 ^a^ |
| RBCs in OR (y/n) | 1.1 | (0.9, 1.4) | 0.24 |
| RBCs in OR (ml/kg) | 0.9 | (0.9, 1.0) | 0.05 ^b^ |
| Factor VIIa in OR (y/n) | 1.1 | (0.8, 1.5) | 0.42 |
| Factor VIIa in OR (ml/kg) | 0.2 | (0.0, 2.1) | 0.18 |
| Prior sternotomies | 1.2 | (0.9, 1.6) | 0.12 |
| Number prior sternotomies | 1.1 | (1.0, 1.2) | 0.02 ^a^ |
| Platelets in ICU (y/n) | 1.3 | (1.0, 1.9) | 0.08 ^b^ |
| Platelets in ICU (ml/kg) | 0.9 | (0.9, 1.0) | 0.04 ^a^ |
| Cryoprecipitate in ICU (y/n) | 1.2 | (0.8, 1.7) | 0.30 |
| Cryoprecipitate in ICU (ml/kg) | 0.9 | (0.9, 0.9) | 0.01 ^a^ |
| Plasma in ICU (y/n) | 1.6 | (1.2, 2.3) | 0.01 ^a^ |
| Plasma in ICU (ml/kg) | 0.9 | (0.9, 1.0) | 0.07 ^b^ |
| RBC in ICU (y/n) | 1.2 | (1.0, 1.6) | 0.04 ^a^ |
| RBC in ICU (ml/kg) | 0.9 | (0.9, 1.0) | 0.04 ^a^ |
| *^a^ Statistically significant*  *^b^ Trend towards statistical significance.*  ^*^ *Combined platelets refers to platelets and concentrated platelets combined for analysis.*  *ICU: intensive care unit; IV: intravenous; kg: kilogram; ml: milliliters; n: no; NOAC: Novel Oral Anticoagulants; OR: operating room; RBC: red blood cells; STAT: Society of Thoracic Surgeons- European Association of Cardio-Thoracic Surgery Congenital Heart Surgery Mortality Categories; y: yes.* | | | |

**Supplemental Table 3. Univariate cox regression of factors and intensive care unit length of stay**

| **Parameter** | **Hazards Ratio** | **95% Confidence Interval** | ***P* value** |
| --- | --- | --- | --- |
| Thrombosis (y/n) | 2.1 | (1.5, 2.8) | <0.001 ^a^ |
| Age (months) | 1.0 | (1.0, 1.0) | <0.001 ^a^ |
| STAT mortality category 4 and 5 | 1.1 | (0.9, 1.4) | 0.38 |
| Chromosomal abnormality | 1.2 | (0.9, 1.6) | 0.31 |
| Cardiopulmonary bypass (min) | 1.0 | (0.9, 1.0) | 0.22 |
| Single Ventricle (y/n) | 1.4 | (0.9, 1.9) | 0.07 ^b^ |
| Prior stroke | 1.3 | (0.9, 2.0) | 0.16 |
| Prior thrombosis | 2.0 | (1.3, 3.1) | <0.001 ^a^ |
| Overall Hypercoagulable state (y/n) | 1.6 | (1.1, 2.4) | 0.01 ^a^ |
| Overall preoperative anticoagulant | 1.4 | (1.1, 1.7) | 0.01 ^a^ |
| Overall preoperative NOAC | 0.6 | (0.3, 1.0) | 0.05 ^b^ |
| Preoperative aspirin | 1.1 | (0.9, 1.5) | 0.24 |
| Preoperative clopidogrel | 0.9 | (0.4, 2.1) | 0.97 |
| Preoperative enoxaparin sodium | 1.1 | (0.7, 1.7) | 0.68 |
| Preoperative coumadin | 0.6 | (0.4, 0.9) | 0.02 ^a^ |
| Preoperative IV Heparin | 3.3 | (2.2, 4.9) | <0.001 ^a^ |
| Preoperative dabigatran | 0.8 | (0.7, 0.9) | 0.002 ^a^ |
| Preoperative rivaroxaban | 0.3 | (0.3, 0.4) | <0.001 ^a^ |
| Preoperative hepatic dysfunction | 1.2 | (0.5, 2.9) | 0.60 |
| Preoperative renal dysfunction | 2.0 | (1.1, 3.6) | 0.02 ^a^ |
| Platelets in OR (y/n) | 1.7 | (1.3, 2.3) | 0.001 |
| Platelets in OR (ml/kg) | 0.9 | (0.9, 0.9) | <0.001 ^a^ |
| Cryoprecipitate in OR (y/n) | 1.3 | (1.1, 1.7) | 0.01 ^a^ |
| Cryoprecipitate in OR (ml/kg) | 1.0 | (1.0, 1.0) | <0.001 ^a^ |
| Cell Saver in OR (Y/N) | 0.9 | (0.6, 1.2) | 0.42 |
| Cell Saver in OR (ml/kg) | 0.9 | (0.9, 0.9) | <0.001 ^a^ |
| RBCs in OR (y/n) | 1.1 | (0.8, 1.4) | 0.49 |
| RBCs in OR (ml/kg) | 0.9 | (0.9, 1.0) | 0.14 |
| Factor VII in OR (y/n) | 1.1 | (0.8, 1.5) | 0.42 |
| Factor VII in OR (ml/kg) | 0.1 | (0.1, 0.9) | 0.04 ^a^ |
| Prior sternotomies | 1.3 | (1.0, 1.6) | 0.08 ^b^ |
| Number prior sternotomies | 1.1 | (1.0, 1.2) | 0.04 ^a^ |
| Platelets in ICU (y/n) | 1.4 | (1.0, 2.1) | 0.07 ^b^ |
| Platelets in ICU (ml/kg) | 1.0 | (0.9, 1.0) | 0.04 ^a^ |
| Cryoprecipitate in ICU (y/n) | 1.2 | (0.8, 1.8) | 0.26 |
| Cryoprecipitate in ICU (ml/kg) | 0.9 | (0.9, 0.9) | 0.02 ^a^ |
| Plasma in ICU (y/n) | 1.7 | (1.1, 2.3) | 0.01 ^a^ |
| Plasma in ICU (ml/kg) | 0.9 | (0.9, 1.0) | 0.05 ^b^ |
| RBC in ICU (y/n) | 1.3 | (1.1, 1.6) | 0.01 ^a^ |
| RBC in ICU (ml/kg) | 0.9 | (0.9, 1.0) | 0.04 ^a^ |
| *^a^ Statistically significant*  *^b^ Trend towards statistical significance.*  *ICU: intensive care unit; IV: intravenous; kg: kilogram; ml: milliliters; n: no; NOAC: Novel Oral Anticoagulants; OR: operating room; RBC: red blood cells; STAT: Society of Thoracic Surgeons- European Association of Cardio-Thoracic Surgery Congenital Heart Surgery Mortality Categories; y: yes.* | | | |

**Supplemental Table 4. Univariate cox regression of factors and ventilation time**

| **Parameter** | **Hazards Ratio** | **95% Confidence Interval** | ***P* value** |
| --- | --- | --- | --- |
| Thrombosis (y/n) | 1.8 | (1.2, 2.7) | 0.005 ^a^ |
| Age (months) | 1.0 | (1.0, 1.0) | <0.001 a |
| STAT mortality category 4 and 5 | 1.1 | (0.8, 1.3) | 0.57 |
| Chromosomal abnormality | 1.2 | (0.9, 1.7) | 0.22 |
| Cardiopulmonary bypass (min) | 1.0 | (1.0, 1.0) | 0.04 ^a^ |
| Single Ventricle (y/n) | 1.3 | (0.8, 2.1) | 0.26 |
| Prior stroke | 1.3 | (0.9, 2.1) | 0.20 |
| Prior thrombosis | 1.6 | (1.0, 2.5) | 0.04 ^a^ |
| Overall Hypercoagulable state (y/n) | 1.6 | (1.0, 2.4) | 0.03 ^a^ |
| Overall preoperative anticoagulant | 1.2 | (0.9, 1.5) | 0.17 |
| Overall preoperative NOAC | 0.2 | (0.1, 0.6) | 0.004 ^a^ |
| Preoperative aspirin | 1.1 | (0.8, 1.4) | 0.59 |
| Preoperative clopidogrel | 0.8 | (0.2, 2.8) | 0.73 |
| Preoperative enoxaparin sodium | 0.9 | (0.6, 1.6) | 0.90 |
| Preoperative coumadin | 0.6 | (0.3, 1.1) | 0.11 |
| Preoperative IV Heparin | 2.6 | (1.9, 3.6) | <0.001 ^a^ |
| Preoperative dabigatran | 0.4 | (0.3, 0.5) | <0.001 ^a^ |
| Preoperative rivaroxaban | 0.1 | (0.0, 0.1) | <0.001 ^a^ |
| Preoperative hepatic dysfunction | 1.4 | (0.5, 3.4) | 0.51 |
| Preoperative renal dysfunction | 2.2 | (1.2, 4.1) | 0.01 ^a^ |
| Platelets in OR (y/n) | 1.4 | (1.0, 1.9) | 0.06 ^b^ |
| Platelets in OR (ml/kg) | 1.0 | (1.0, 1.0) | 0.002 ^a^ |
| Cryoprecipitate in OR (y/n) | 1.2 | (0.9, 1.4) | 0.21 |
| Cryoprecipitate in OR (ml/kg) | 1.0 | (1.0, 1.0) | <0.001 ^a^ |
| Cell Saver in OR (y/n) | 0.9 | (0.7, 1.3) | 0.62 |
| Cell Saver in OR (ml/kg) | 1.0 | (1.0, 1.0) | 0.001 ^a^ |
| RBCs OR in (y/n) | 1.0 | (0.7, 1.3) | 0.86 |
| RBCs OR in (ml/kg) | 1.0 | (1.0, 1.0) | 0.60 |
| Factor VIIa in OR (y/n) | 0.9 | (0.7, 1.2) | 0.46 |
| Factor VIIa in OR (ml/kg) | 1.3 | (0.2, 8.0) | 0.78 |
| Prior sternotomies | 1.2 | (0.9, 1.5) | 0.28 |
| Number prior sternotomies | 1.1 | (1.0, 1.2) | 0.009 ^a^ |
| Platelets in ICU (y/n) | 1.5 | (1.0, 2.1) | 0.03 ^a^ |
| Platelets in ICU (ml/kg) | 1.0 | (1.0, 1.0) | 0.02 ^a^ |
| Cryoprecipitate in ICU (y/n) | 1.4 | (0.9, 1.9) | 0.09 ^b^ |
| Cryoprecipitate in ICU (ml/kg) | 1.0 | (0.9, 1.0) | 0.002 ^a^ |
| Plasma in ICU (y/n) | 1.6 | (1.1, 2.2) | 0.006 ^a^ |
| Plasma in ICU (ml/kg) | 1.0 | (1.0, 1.0) | 0.02 ^a^ |
| RBC in ICU (y/n) | 1.5 | (1.2, 1.9) | <0.001 ^a^ |
| RBC in ICU (ml/kg) | 1.0 | (1.0, 1.0) | 0.03 ^a^ |
| *^a^ Statistically significant*  *^b^ Trend towards statistical significance.*  *ICU: intensive care unit; IV: intravenous; kg: kilogram; ml: milliliters; n: no; NOAC: Novel Oral Anticoagulants; OR: operating room; RBC: red blood cells; STAT: Society of Thoracic Surgeons- European Association of Cardio-Thoracic Surgery Congenital Heart Surgery Mortality Categories; y: yes.* | | | |

**Supplemental Table 5. Multivariable cox regression of factors associated with longer hospital and intensive care unit length of stay (Harrell’s c-index=0.67, 0.68, respectively)**

| **Parameter** | **Hospital LOS** | | **ICU LOS** | |
| --- | --- | --- | --- | --- |
|  | **HR (95% CI)** | ***P* value** | **HR (95% CI)** | ***P* Value** |
| Thrombosis (y/n) | 2.3 (1.5, 3.4) | <0.001 ^a^ | 2.6 (1.7, 3.9) | <0.001 ^a^ |
| Age (months) | 1.0 (1.0, 1.0) | <0.001 ^a^ | 1.0 (1.0, 1.0) | <0.001 ^a^ |
| Prior history of thrombosis | 2.7 (1.7, 4.2) | <0.001 ^a^ | 2.7 (1.7, 4.2) | <0.001 ^a^ |
| *^a^ Statistically significant  CI: confidence interval; HR: hazards ratio; ICU: intensive care unit; n: no; LOS: length of stay; y: yes* | | | | |

**Supplemental Table 6. Multivariable cox regression of factors associated with longer ventilation time (Harrell’s c-index=0.66)**

| **Parameters** | **Hazard Ratio** | **95% CI** | ***P* value** |
| --- | --- | --- | --- |
| Thrombosis | 1.8 | (1.2, 2.8) | 0.007 ^a^ |
| Age (months) | 1.0 | (1.0, 1.0) | <0.001 ^a^ |
| RBC ICU (y/n) | 1.5 | (1.2, 1.9) | <0.001 ^a^ |
| *^a^ Statistically significant*  *CI: confidence interval; HR: hazards ratio; ICU: intensive care unit; n: no; RBC: red blood cells; y: yes* | | | |

**Supplemental Table 7. Univariate median regression of factors and hospital cost**

| **Parameter** | **Estimate** | **95% Confidence Interval** | ***P* value** |
| --- | --- | --- | --- |
| Thrombosis (y/n) | 157,291 | (-81,372, 395,955) | 0.19 |
| Late stage thrombosis | 516,522 | (175,809, 857,235) | 0.003 ^a^ |
| Age (months) | -133.9 | (-225.8, -42.2) | 0.004 ^a^ |
| Time to diagnosis category | 161,604 | (32,279, 290,929) | 0.01 ^a^ |
| STAT mortality category 4 and 5 | 37,787 | (6,778, 68,797) | 0.01 ^a^ |
| Chromosomal abnormality | 40,689 | (-9,990, 91,368) | 0.11 |
| Cardiopulmonary bypass (min) | 230.75 | (47.89, 413.61) | 0.01 ^a^ |
| Single Ventricle (y/n) | 41,097 | (-29,715, 111,911) | 0.25 |
| Prior stroke | 39,143 | (-60,249, 138,536) | 0.44 |
| Prior thrombosis | 163,774 | (-12,550, 340,099) | 0.07 ^b^ |
| Overall Hypercoagulable state (y/n) | 159,084 | (-144,847, 463,016) | 0.30 |
| Overall preoperative anticoagulant | 43,639 | (7,612, 79,666) | 0.02 ^a^ |
| Overall preoperative NOAC | 56,787 | (-3,221,246, 3,334,821) | 0.97 |
| Preoperative aspirin | 38,983 | (28.07, 77,938) | 0.04 ^a^ |
| Preoperative clopidogrel | 134,891 | (-554,099, 823,881) | 0.70 |
| Preoperative enoxaparin sodium | 37,062 | (-99,585, 173,709) | 0.59 |
| Preoperative coumadin | -1,518 | (-132,410, 129,372) | 0.98 |
| Preoperative IV Heparin | 534,804 | (246,164, 823,444) | <0.001 ^a^ |
| Preoperative dabigatran | 56,787 | (-10,534,347, 10,647,923) | 0.99 |
| Preoperative rivaroxaban | -36,100 | (-9,383,878, 9,311,677) | 0.99 |
| Preoperative hepatic dysfunction | -38,505 | (-299,059, 222,047) | 0.77 |
| Preoperative renal dysfunction | 110,166 | (-365,198, 585,532) | 0.64 |
| Platelets in OR (y/n) | 75,033 | (46,743, 103,323) | <0.001 ^a^ |
| Platelets in OR (ml/kg) | 1,838 | (653.3, 3,023.5) | 0.003 ^a^ |
| Cryoprecipitate in OR (y/n) | 59,198 | (34,871, 83,525) | <0.001 ^a^ |
| Cryoprecipitate in OR (ml/kg) | -2.07 | (-503.8, 499.7) | 0.99 |
| Cell Saver in OR (y/n) | -24,698 | (-110,340, 60,944) | 0.57 |
| Cell Saver in OR (ml/kg) | 1,018 | (-1.0098, 2,038) | 0.05 ^b^ |
| RBCs in OR (y/n) | 69,959 | (8,764, 131,155) | 0.02 ^a^ |
| RBCs in OR (ml/kg) | 1,561 | (-644, 3,767) | 0.16 |
| Factor VIIa in OR (y/n) | 66,385 | (12,529, 120,240) | 0.01 ^a^ |
| Factor VIIa in OR (ml/kg) | 324,209 | (-1,725,436, 2,373,855) | 0.76 |
| Prior sternotomies | 21,523 | (-26,755, 69,802) | 0.38 |
| Number prior sternotomies | -759.4 | (-19,324, 17,805) | 0.93 |
| Platelets in ICU (y/n) | 32,944 | (-81,784, 147,673) | 0.57 |
| Platelets in ICU (ml/kg) | 3,844 | (-655.97, 8,345.8) | 0.09 ^b^ |
| Cryoprecipitate in ICU (y/n) | 34,591 | (-69,530, 138,713) | 0.51 |
| Cryoprecipitate in ICU (ml/kg) | 3,101 | (-5,565, 11,767) | 0.48 |
| Plasma in ICU (y/n) | 34,591 | (-78,029, 147,211) | 0.54 |
| Plasma in ICU (ml/kg) | 2,292 | (-612.2, 5,197) | 0.12 |
| RBC in ICU (y/n) | 34,098 | (-87.7, 68,284.5) | 0.05 ^b^ |
| RBC in ICU (ml/kg) | 1,710 | (208.6, 3,213) | 0.03 ^a^ |
| *^a^ Statistically significant*  *^b^ Trend towards statistical significance.*  *ICU: intensive care unit; IV: intravenous; kg: kilogram; ml: milliliters; n: no; NOAC: Novel Oral Anticoagulants; OR: operating room; RBC: red blood cells; STAT: Society of Thoracic Surgeons- European Association of Cardio-Thoracic Surgery Congenital Heart Surgery Mortality Categories; y: yes.* | | | |

**Supplemental Figure 1: Flowchart of patient selection.** A total of 334 patients with postoperative hyperfibrinogenemia (>400 mg/dL) were included: of these, 306 patients did not have postoperative thrombosis while 28 patients did have postoperative thrombosis. *mg: milligram; dL: deciliter.*

**
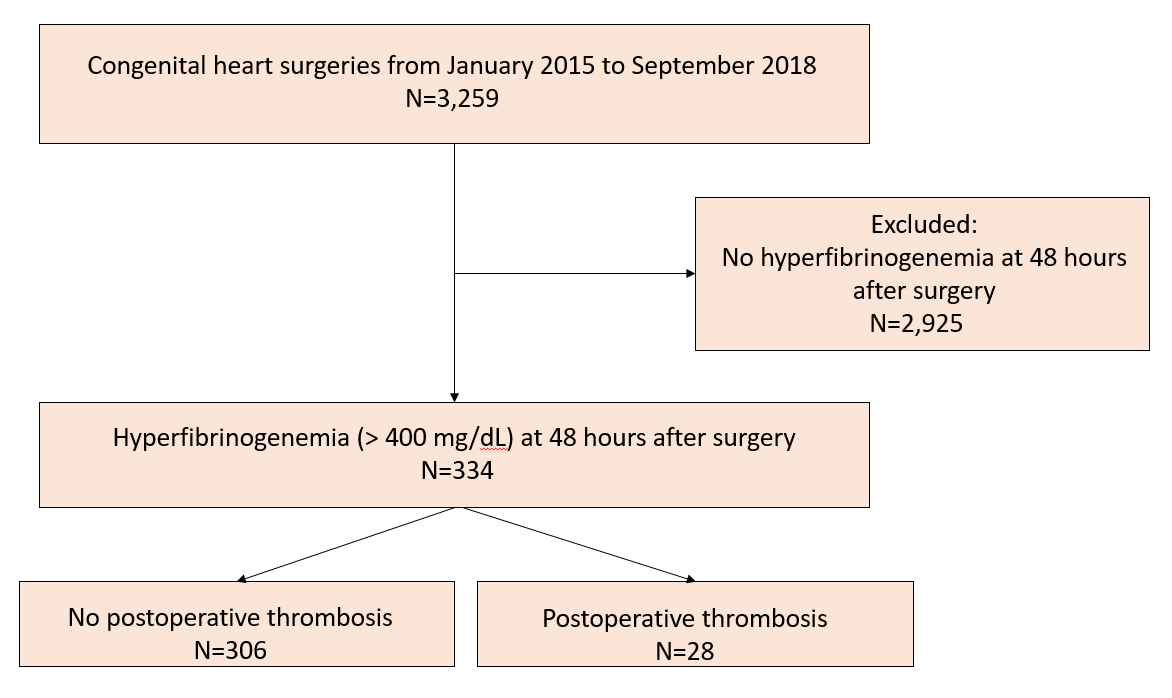
**
